# Clinicopathological Predictors of Pathological Response and Survival in Older Patients Undergoing Perioperative FLOT Chemotherapy for Resectable Gastric and Gastroesophageal Junction Adenocarcinoma

**DOI:** 10.3390/medicina62081612

**Published:** 2026-08-21

**Authors:** Aykut Özmen, Tuba Tahtalı, Gündüz Karaoğlan, Mehmet Kutlay, Ulviye Oflas, Tanju Kapağan, Nilüfer Bulut, Gökmen Umut Erdem

**Affiliations:** Department of Medical Oncology, Başakşehir Çam and Sakura City Hospital, 34480 Istanbul, Turkey; dr.tubatahtali@gmail.com (T.T.); gunduzkaraoglan@gmail.com (G.K.); dr_kutlay21@hotmail.com (M.K.); ulvyeoflas@hotmail.com (U.O.); tanjukapagan2016@gmail.com (T.K.); ferlur@gmail.com (N.B.); gokmenumut@hotmail.com (G.U.E.)

**Keywords:** gastric cancer, older adults, FLOT, perioperative chemotherapy, pathological response, prognostic factors, disease-free survival, overall survival

## Abstract

*Background and Objectives*: Evidence regarding predictors of pathological response and long-term outcomes in older patients receiving perioperative FLOT chemotherapy for resectable gastric or gastroesophageal junction cancer remains limited. We aimed to identify factors associated with pathological response and survival in patients aged ≥65 years treated with neoadjuvant FLOT. *Materials and Methods*: This retrospective single-center study included 86 consecutive patients (median age, 69 years; 72.1% male) aged ≥65 years with resectable gastric or gastroesophageal junction adenocarcinoma who underwent neoadjuvant FLOT chemotherapy followed by curative-intent gastrectomy. Major pathological response was defined as College of American Pathologists tumor regression grade (CAP TRG) 0–1. Logistic regression analyses were performed to identify predictors of pathological response. Disease-free survival (DFS) and overall survival (OS) were analyzed using the Kaplan–Meier method and Cox proportional hazards regression analyses. *Results*: Overall, 14 patients (16.3%) achieved a major pathological response. Body mass index (BMI) ≥ 25 kg/m^2^ (OR 5.13, *p* = 0.04) was independently associated with a major pathological response. During a median follow-up of 29.2 months, 39 patients (45.3%) developed recurrence and 35 (40.7%) died. ECOG performance status (ECOG PS) ≥ 1 was independently associated with both inferior DFS (HR = 2.76, *p* = 0.02) and OS (HR = 2.52, *p* = 0.045). In addition, ypN stage 2–3 (HR = 2.58, *p* = 0.008) was independently associated with worse DFS, whereas positive surgical margins were independently associated with worse OS (HR = 2.62, *p* = 0.02). *Conclusions*: In older patients with resectable gastric or gastroesophageal junction adenocarcinoma treated with perioperative FLOT chemotherapy, baseline BMI was independently associated with major pathological response, whereas ECOG PS and postoperative pathological factors were independently associated with survival. These findings highlight the importance of careful patient selection and individualized multimodal treatment in this population.

## 1. Introduction

Gastric cancer remains one of the leading causes of cancer-related mortality worldwide despite continuous advances in multimodal treatment strategies [1]. For patients with locally advanced resectable gastric and gastroesophageal junction adenocarcinoma, perioperative chemotherapy combined with curative-intent surgery has become the standard treatment, significantly improving disease-free and overall survival compared with surgery alone. The FLOT regimen (fluorouracil, leucovorin, oxaliplatin, and docetaxel) demonstrated superior survival outcomes over previous perioperative chemotherapy regimens and is currently recommended by international guidelines for medically fit patients with resectable disease [2,3].

As the global population ages, older adults constitute an increasing proportion of patients diagnosed with gastric cancer. However, older patients remain substantially underrepresented in prospective clinical trials evaluating perioperative chemotherapy. Consequently, treatment decisions in this population are frequently individualized because chronological age alone does not adequately reflect physiological reserve, treatment tolerance, or long-term prognosis. Comprehensive geriatric assessment and functional status have therefore emerged as key determinants of treatment selection and clinical outcomes in older adults with cancer [4]. Nevertheless, evidence regarding prognostic factors and treatment outcomes among older patients receiving contemporary perioperative FLOT remains limited.

Pathological tumor regression following neoadjuvant chemotherapy has emerged as an important surrogate marker of treatment efficacy and has been associated with improved long-term survival in patients with resectable gastric cancer [5]. Furthermore, several postoperative pathological factors, including pathological nodal stage, lymphovascular invasion, perineural invasion, and resection margin status, have consistently demonstrated independent prognostic value following curative gastrectomy [6]. Despite these observations, relatively few studies have comprehensively evaluated pretreatment clinical characteristics, pathological response, postoperative pathological factors, and completion of adjuvant chemotherapy within an exclusively older cohort treated with modern perioperative FLOT.

Therefore, this study aimed to identify pretreatment clinical factors associated with favorable pathological response following neoadjuvant FLOT chemotherapy and to determine independent prognostic factors for disease-free and overall survival in patients aged 65 years or older with resectable gastric or gastroespohageal junction cancer.

## 2. Materials and Methods

### 2.1. Study Design and Patients

This retrospective single-center cohort study included consecutive patients aged 65 years or older with histologically confirmed, resectable gastric or gastroesophageal junction cancer who received neoadjuvant FLOT (fluorouracil, leucovorin, oxaliplatin, and docetaxel) chemotherapy and subsequently underwent curative-intent gastrectomy at Basaksehir Cam and Sakura City Hospital between June 2020 and December 2025. Patients with metastatic disease at diagnosis, previous gastric malignancy, or incomplete clinicopathological or follow-up data were excluded. Initial staging was performed using upper gastrointestinal endoscopy with biopsy and contrast-enhanced thoracoabdominal computed tomography. Additional endoscopic ultrasonography (EUS), positron emission tomography/computed tomography (PET/CT), and diagnostic laparoscopy were performed when clinically indicated. Clinical and pathological staging were determined according to the 8th edition of the American Joint Committee on Cancer (AJCC) TNM staging system [7].

Patients were treated with the standard FLOT regimen consisting of docetaxel (50 mg/m^2^), oxaliplatin (85 mg/m^2^), leucovorin (200 mg/m^2^), and 5-fluorouracil (2600 mg/m^2^ administered as a 24 h continuous infusion) every two weeks. Four preoperative cycles were planned whenever feasible. Following curative-intent gastrectomy, four additional postoperative FLOT cycles were recommended according to the patient’s postoperative recovery, performance status, and treatment tolerance. Dose reductions, treatment delays, or treatment discontinuation were performed at the discretion of the treating physician according to routine institutional practice, taking into account treatment-related toxicities, performance status, laboratory parameters, and overall clinical condition. The timing of surgery was determined at multidisciplinary team meetings following completion of neoadjuvant chemotherapy.

The study protocol for retrospective data collection and analysis was approved by the Institutional Ethics Committee of Başakşehir Çam and Sakura City Hospital (Protocol Number: KAEK/22.04.2026.07), and the study was conducted in accordance with the ethical principles of the Declaration of Helsinki.

### 2.2. Data Collection and Pathological Assessment

Demographic, clinical, pathological, treatment, and follow-up data were retrospectively retrieved from the institutional electronic medical records. Baseline demographic and clinical variables included age, sex, Eastern Cooperative Oncology Group performance status (ECOG PS), body mass index (BMI), tumor location, histological subtype, tumor grade, clinical TNM stage, carcinoembryonic antigen (CEA), HER2 status, microsatellite instability (MSI) status, and the interval between completion of neoadjuvant chemotherapy and surgery. Mismatch repair (MMR) status was routinely assessed by immunohistochemical evaluation of MLH1, PMS2, MSH2, and MSH6 proteins, and tumors were subsequently classified as MSI-high or MSI-low/MSS according to routine institutional pathological practice. Surgical and pathological variables included the type of gastrectomy, surgical margin status, pathological TNM stage, lymphovascular invasion (LVI), perineural invasion (PNI), and receipt of adjuvant chemotherapy.

All resection specimens were evaluated by two experienced gastrointestinal pathologists as part of routine institutional pathological practice. No central pathology review was performed. Tumor regression was assessed using the College of American Pathologists (CAP) Tumor Regression Grade (TRG) system, in which TRG 0 indicates complete response (no viable residual tumor cells), TRG 1 near-complete response (single cells or rare small groups of residual cancer cells), TRG 2 partial response (residual cancer with evident tumor regression), and TRG 3 poor or no response (extensive residual cancer with no evident tumor regression). For statistical analyses, patients were classified as having either a major pathological response (CAP TRG 0–1) or a minor pathological response (CAP TRG 2–3). This grouping was selected because CAP TRG 0 and 1 represent complete or near-complete tumor regression and broadly correspond to the major responder categories in other commonly used tumor regression grading systems, such as the Becker and Mandard classifications. Pathological staging was performed according to the 8th edition of the American Joint Committee on Cancer (AJCC) TNM staging system, and tumor regression grading was assessed according to the CAP protocol [7,8].

### 2.3. Follow-Up and Study Endpoints

Following curative-intent gastrectomy, patients were routinely followed according to institutional surveillance protocols with regular clinical evaluations, laboratory investigations, and radiological imaging. Follow-up visits were generally scheduled every three months during the first two postoperative years, every six months until five years, and annually thereafter, or earlier if clinically indicated.

The primary endpoints of the study were disease-free survival (DFS) and overall survival (OS). DFS was defined as the interval from the date of surgery to the first documented disease recurrence or death from any cause, whichever occurred first. OS was defined as the interval from the date of surgery to death from any cause. Patients without recurrence or death were censored at the date of last follow-up for DFS, and patients alive at last follow-up were censored at the date of last follow-up for OS. The secondary endpoint was to identify pretreatment clinical factors associated with major pathological response (CAP TRG 0–1) following neoadjuvant FLOT chemotherapy.

### 2.4. Statistical Analysis

Continuous variables were expressed as median (range), whereas categorical variables were presented as frequencies and percentages. Comparisons between patients with major pathological response (CAP TRG 0–1) and minor pathological response (CAP TRG 2–3) were performed using the Mann–Whitney U test for continuous variables and the chi-square test or Fisher’s exact test, as appropriate, for categorical variables. BMI was evaluated both as a continuous variable and as a categorical variable using a cutoff of 25 kg/m^2^, corresponding to the World Health Organization (WHO) definition of overweight. Factors associated with major pathological response were initially evaluated using univariable binary logistic regression analysis. Given the limited number of major pathological response events (*n* = 14), the number of covariates was deliberately restricted to reduce the risk of overfitting. Only BMI and clinical stage, which were significantly associated with major pathological response in the univariable analyses, were included simultaneously in the multivariable binary logistic regression model, corresponding to approximately seven outcome events per included variable. Odds ratios (ORs) were reported with corresponding 95% confidence intervals (95% CIs).

DFS and OS were estimated using the Kaplan–Meier method and compared using the log-rank test. Prognostic factors associated with DFS and OS were evaluated using univariable and multivariable Cox proportional hazards regression analyses. Variables associated with the outcomes in univariable analyses were considered as candidates for the multivariable Cox proportional hazards regression models; however, univariable statistical significance was not used as the sole criterion for inclusion. Given the limited number of outcome events, the final models were deliberately restricted to a parsimonious set of variables selected on the basis of statistical evidence, clinical relevance, and the number of outcome events to reduce the risk of overfitting. The proportional hazards assumption was assessed using Schoenfeld residuals (global and variable-specific tests) and was satisfied for all final multivariable models. Hazard ratios (HRs) were reported with corresponding 95% CIs.

All statistical analyses were performed using IBM SPSS Statistics for Windows, version 27.0 (IBM Corp., Armonk, NY, USA). Kaplan–Meier survival curves were generated using R software (version 4.5.2; R Foundation for Statistical Computing, Vienna, Austria). All tests were two-sided, and a *p* value <0.05 was considered statistically significant.

## 3. Results

### 3.1. Patient Characteristics

**Figure 1:**
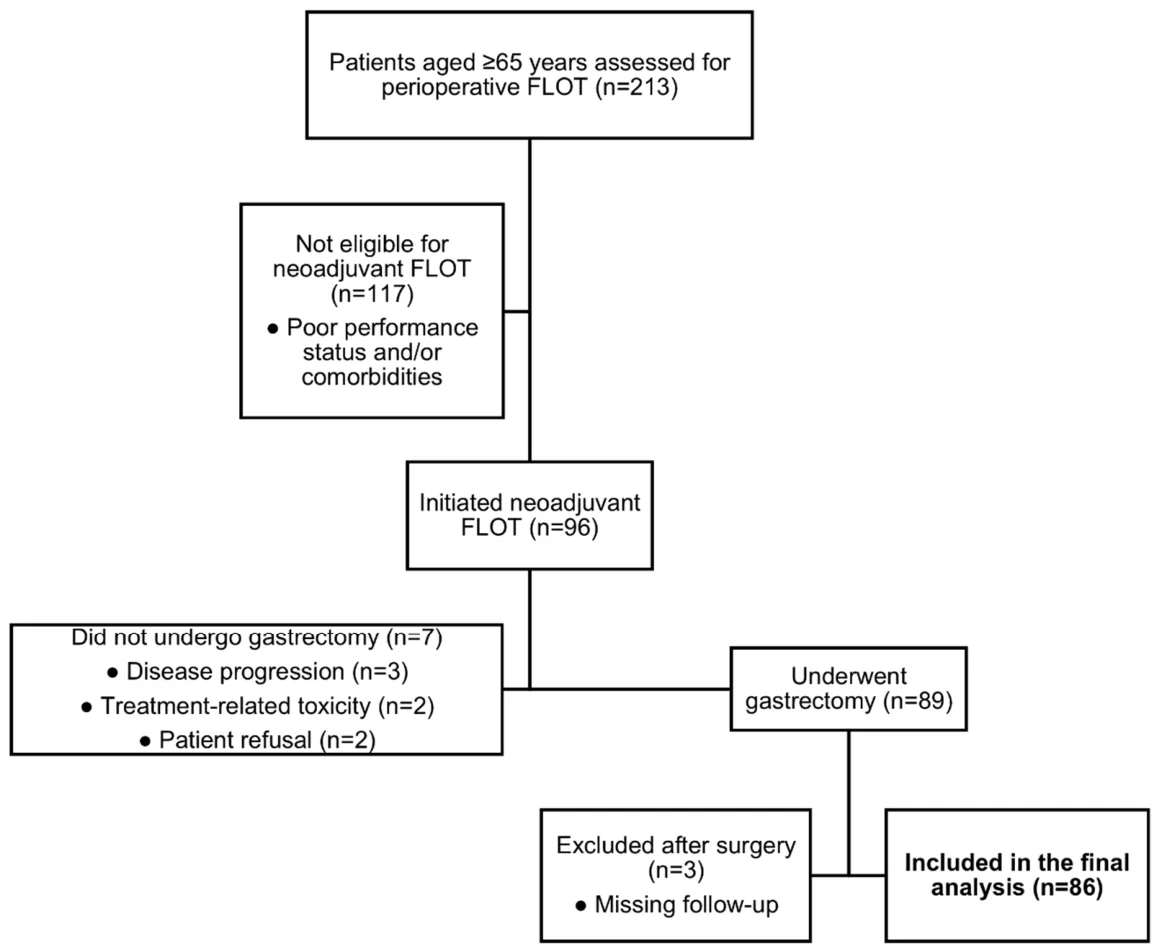
Patient flow diagram. Flow of patients aged ≥65 years assessed for perioperative FLOT during the study period. The diagram summarizes patient selection, reasons for exclusion before and after gastrectomy, and derivation of the final study cohort included in the analysis.

A total of 86 consecutive patients aged 65 years or older who received neoadjuvant FLOT chemotherapy followed by curative-intent gastrectomy were included in the study. The patient selection process is illustrated in the STROBE-style flow diagram (Figure 1). The median age was 69 years (range, 65–78), and 62 patients (72.1%) were male. Most patients had an ECOG PS of ≥1 (59.3%), clinical stage III disease (79.1%), and clinically node-positive disease (87.2%). The median interval between the last cycle of neoadjuvant chemotherapy and surgery was 48 days (range, 16–124). Overall, 80 patients (93%) completed the planned four cycles of neoadjuvant FLOT chemotherapy, whereas six patients received a different number of preoperative cycles. Curative (R0) resection was achieved in 70 patients (81.4%), and 69 patients (80.2%) received postoperative adjuvant chemotherapy. The baseline demographic, clinical, treatment-related, and pathological characteristics of the study cohort are summarized in Table 1 and Table 2.

### 3.2. Factors Associated with Pathological Response

Among the 86 patients included in the final analysis, CAP tumor regression grades were distributed as follows: TRG 0 in 9 patients (10.5%), TRG 1 in 5 (5.8%), TRG 2 in 47 (54.7%), and TRG 3 in 25 (29.1%). Overall, 14 patients (16.3%) achieved a major pathological response (CAP TRG 0–1), whereas 72 (83.7%) had a minor pathological response (CAP TRG 2–3). Patients with a major pathological response more frequently had a BMI ≥ 25 kg/m^2^ (85.7% vs. 52.8%, *p* = 0.02), lower clinical T stage (*p* = 0.002), lower clinical stage (*p* = 0.03), and different primary tumor location (*p* = 0.04) compared with those with a minor pathological response (Table 1).

Patients with a major pathological response also demonstrated more favorable postoperative pathological findings, including lower ypT stage (*p* < 0.001), lower ypN stage (*p* = 0.001), lower pathological stage (*p* < 0.001), absence of LVI (*p* < 0.001) and PNI (*p* < 0.001), and a higher R0 resection rate (*p* < 0.001). Adjuvant chemotherapy administration did not differ significantly between the groups (*p* = 0.19) (Table 2).

Among the pretreatment variables, only BMI and clinical stage were significantly associated with major pathological response in the univariable logistic regression analyses. In the multivariable model, BMI ≥ 25 kg/m^2^ remained independently associated with a major pathological response (OR 5.13, 95% CI 1.05–25.19; *p* = 0.04), whereas clinical stage showed borderline statistical significance (OR 3.54, 95% CI 0.99–12.69; *p* = 0.05) (Table 3).

### 3.3. Survival Outcomes

During a median follow-up of 29.2 months (95% CI, 24.8–33.5), 39 patients (45.3%) experienced disease recurrence and 35 patients (40.7%) died. Median DFS was 35.6 months (95% CI, 14–57.2) and median OS was 37.4 months (95% CI, 24.5–50.3).

**Figure 2:**
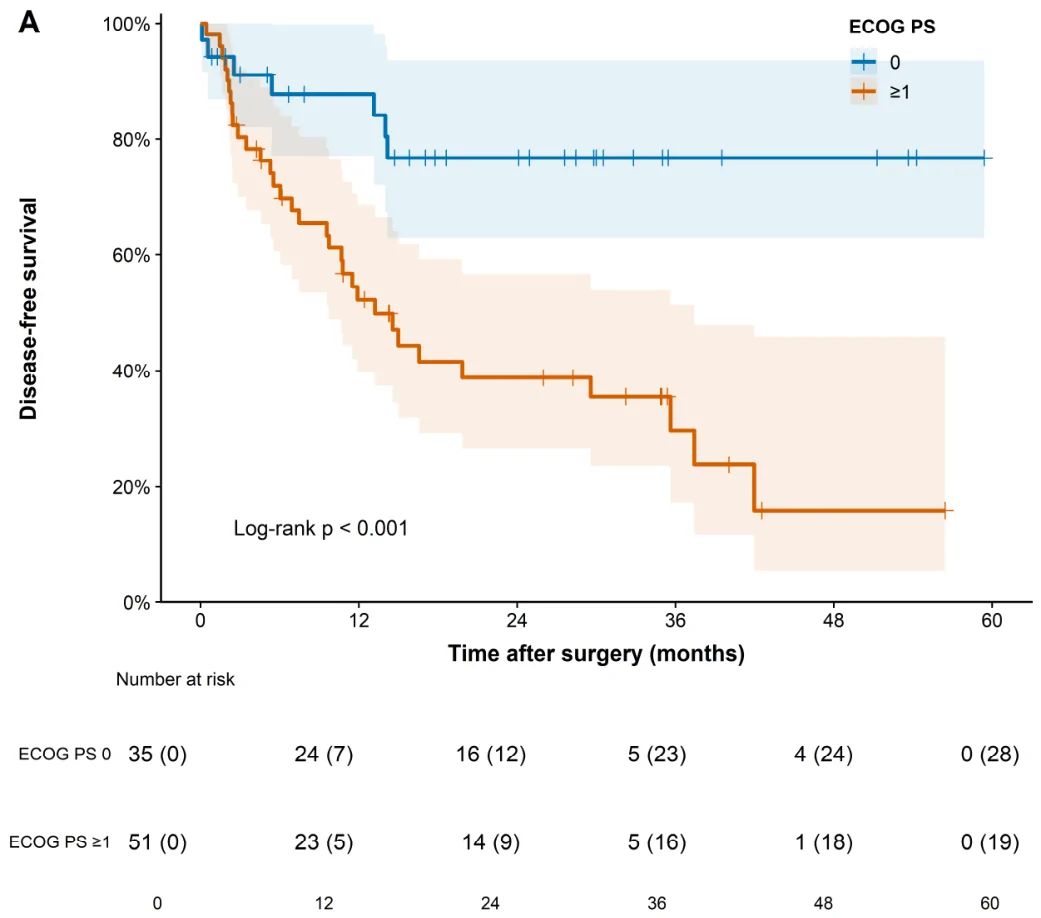

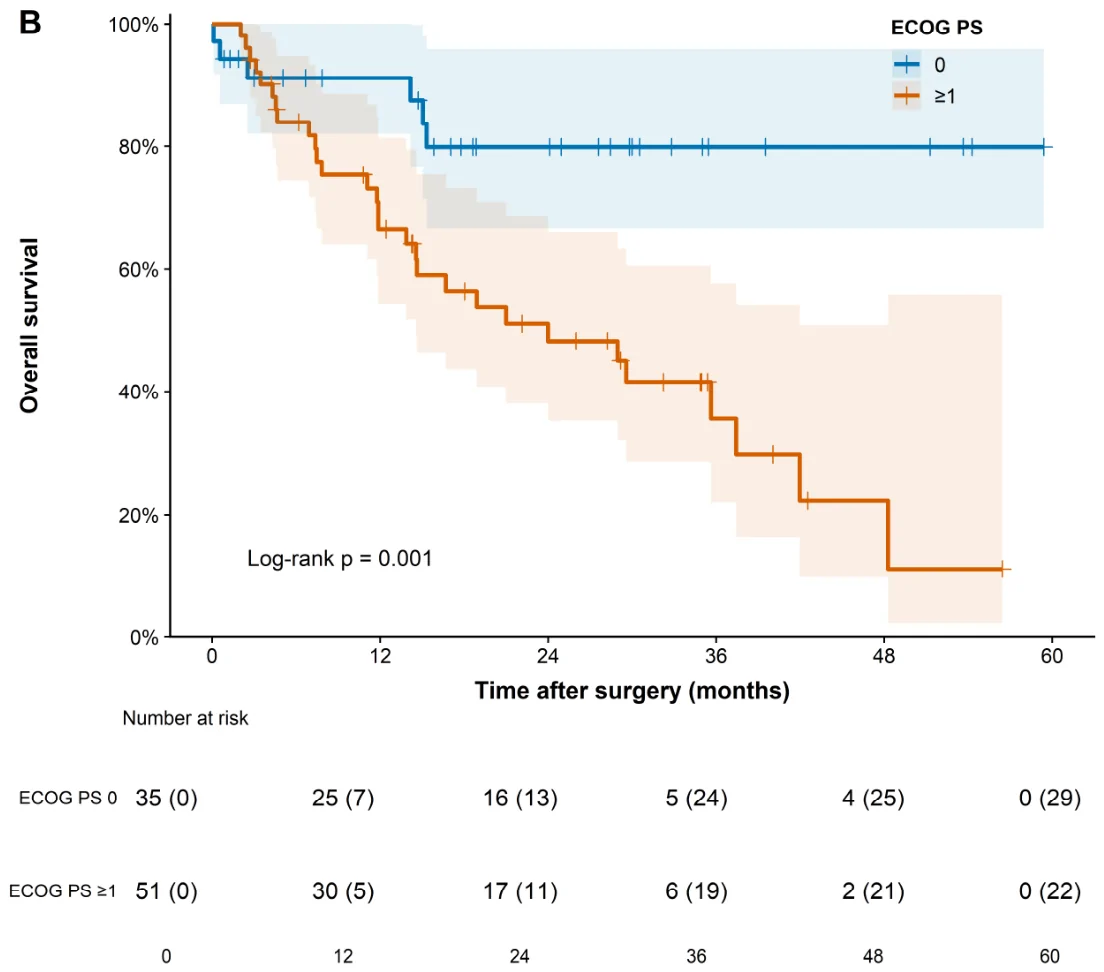
Kaplan–Meier survival curves according to baseline ECOG performance status: (**A**) disease-free survival and (**B**) overall survival. Shaded areas represent 95% confidence intervals, and tick marks indicate censored observations. The numbers below each plot indicate patients at risk, with cumulative numbers censored shown in parentheses. ECOG PS, Eastern Cooperative Oncology Group performance status.

Patients with an ECOG PS of 0 had significantly longer DFS (*p* = 0.001) and OS (*p* = 0.003) than those with an ECOG PS of ≥1 (Figure 2A,B). In addition, several postoperative pathological factors were significantly associated with survival. Patients with ypN0–1 disease had significantly longer DFS than those with ypN2–3 disease (*p* < 0.001), whereas patients who achieved R0 resection had significantly better OS than those with positive surgical margins (*p* < 0.001).

### 3.4. Prognostic Factors for Survival

In the univariable Cox proportional hazards regression analyses for DFS, age ≥ 70 years, ECOG PS ≥ 1, ypT stage 3–4, ypN stage 2–3, LVI, PNI, and positive surgical margins were associated with worse DFS. In the multivariable analysis, ECOG PS ≥ 1 (HR 2.76, 95% CI 1.19–6.40; *p* = 0.02) and ypN stage 2–3 (HR 2.58, 95% CI 1.29–5.17; *p* = 0.008) remained independently associated with inferior DFS (Table 4).

For OS, the univariable analyses identified age ≥ 70 years, ECOG PS ≥ 1, proximal tumor location, grade 3 histology, ypN stage 2–3, LVI, PNI, and positive surgical margins as factors associated with worse OS. In the multivariable model, ECOG PS ≥ 1 (HR 2.52, 95% CI 1.02–6.23; *p* = 0.045) and positive surgical margins (HR 2.62, 95% CI 1.20–5.76; *p* = 0.02) remained independently associated with inferior OS, whereas ypN stage 2–3 showed borderline statistical significance (HR 2.02, 95% CI 0.97–4.22; *p* = 0.06) (Table 5).

## 4. Discussion

In this retrospective cohort of patients aged 65 years or older with resectable gastric or gastroesophageal junction adenocarcinoma treated with neoadjuvant FLOT chemotherapy, we identified several clinically relevant factors associated with pathological response and long-term survival. BMI ≥ 25 kg/m^2^ was independently associated with a major pathological response, whereas ECOG PS was independently associated with both DFS and OS. In addition, ypN stage was independently associated with worse DFS, while positive surgical margins were independently associated with inferior OS. These findings highlight the importance of careful patient selection, optimization of functional status, and consideration of baseline nutritional reserve in older patients undergoing perioperative FLOT chemotherapy.

Our study identified baseline ECOG PS as an independent prognostic factor for both DFS and OS. Patients with an ECOG PS of 0 experienced significantly better long-term survival than those with impaired performance status. Although chronological age is frequently considered during treatment decision-making in older patients with gastric cancer, our findings suggest that functional status may be more informative than age alone in predicting long-term outcomes. This observation is consistent with contemporary geriatric oncology recommendations, which emphasize physiological reserve, functional capacity, and comprehensive geriatric assessment rather than chronological age when selecting older adults for systemic anticancer therapy [4]. Although the GO2 trial was conducted in the palliative setting, it similarly demonstrated that baseline fitness and frailty are important determinants of treatment tolerance and clinical outcomes in older patients with gastroesophageal cancer, supporting an individualized treatment approach based on functional status rather than age alone [9]. Collectively, these findings reinforce the importance of careful functional assessment when selecting older patients for perioperative FLOT chemotherapy.

Despite the increasing use of perioperative FLOT chemotherapy, evidence specifically addressing older patients with resectable gastric cancer remains limited because older individuals have been underrepresented in prospective clinical trials. The FLOT65+ trial demonstrated that perioperative FLOT is feasible in carefully selected older patients; however, only 58.6% of patients completed the planned preoperative treatment, underscoring the challenges of delivering intensive perioperative chemotherapy in this population [10]. In our cohort, 93% of patients completed the planned four preoperative FLOT cycles. This finding should be interpreted cautiously, as our retrospective study included only patients who initiated neoadjuvant FLOT and subsequently underwent gastrectomy, thereby selecting a relatively fitter population. More recent real-world studies have likewise shown that medically fit older patients can achieve survival outcomes comparable to those of younger patients when treated with multimodal therapy, emphasizing that patient fitness rather than chronological age is the principal determinant of treatment benefit [11]. Our findings extend these observations by demonstrating that baseline ECOG PS was independently associated with long-term survival, whereas BMI was independently associated with major pathological response, suggesting that these clinical characteristics may be associated with different aspects of treatment outcome in older patients receiving perioperative FLOT chemotherapy.

Our study also found that BMI ≥ 25 kg/m^2^, corresponding to the WHO definition of overweight, was independently associated with major pathological response. Although BMI is an imperfect surrogate for nutritional status and body composition, a higher BMI may reflect greater physiological reserve in some older patients undergoing intensive multimodal treatment; however, it should not be interpreted as a direct measure of nutritional adequacy. Conversely, a lower BMI may be associated with malnutrition, frailty, and occult sarcopenia, all of which can adversely affect chemotherapy tolerance and treatment completion. This finding is also consistent with the concept of the “obesity paradox,” whereby overweight patients with cancer may experience more favorable outcomes than leaner individuals, possibly because of greater metabolic reserve during systemic treatment. Nevertheless, BMI alone cannot distinguish between adiposity and skeletal muscle mass, nor does it capture edema, cancer-related weight loss, or sarcopenic obesity, all of which may substantially influence treatment tolerance and prognosis. Previous studies have demonstrated that impaired body composition, particularly sarcopenia, is associated with increased chemotherapy toxicity, early treatment discontinuation, and inferior oncological outcomes in patients receiving neoadjuvant treatment for gastric cancer [12]. Furthermore, recent systematic reviews have highlighted the close relationship between nutritional status, body composition, and treatment outcomes in patients undergoing perioperative therapy for gastric cancer [13]. Interestingly, although BMI was independently associated with major pathological response, it was not independently associated with survival outcomes after multivariable adjustment. Moreover, in the univariable survival analyses, higher BMI showed a numerical trend toward poorer DFS and OS, although these associations did not persist after adjustment for other clinicopathological variables. This apparent discrepancy suggests that BMI may be more closely related to treatment tolerance and the likelihood of achieving tumor regression than to long-term prognosis, which is likely determined predominantly by baseline functional status and postoperative pathological factors. Our findings therefore support the importance of routine nutritional assessment before treatment initiation. However, the observed association between BMI and major pathological response should be considered hypothesis-generating and should not be interpreted as establishing BMI as a predictive biomarker or as a criterion for selecting patients for perioperative FLOT chemotherapy.

Postoperative pathological characteristics also provided important prognostic information in our cohort. Advanced ypN stage was independently associated with inferior DFS, whereas positive surgical margins were independently associated with worse OS. These findings are consistent with previous studies demonstrating that residual nodal disease following neoadjuvant chemotherapy is among the strongest prognostic determinants after curative-intent gastrectomy and may reflect persistent systemic tumor burden [14,15]. Likewise, achievement of an R0 resection remains a fundamental objective of curative treatment and has consistently been associated with improved long-term outcomes [15]. Collectively, our results emphasize that postoperative pathological assessment continues to provide essential prognostic information after modern perioperative chemotherapy and may help identify older patients who require closer surveillance and individualized postoperative management.

Despite these findings, several limitations should be acknowledged. First, this was a retrospective single-center study, which may have introduced selection bias and limited the generalizability of our findings. Moreover, only patients who initiated neoadjuvant FLOT and subsequently underwent gastrectomy were included in the final analysis. Patients who were ineligible for neoadjuvant FLOT or did not proceed to surgery because of disease progression, treatment-related toxicity, or patient refusal were excluded, potentially resulting in a relatively fitter study population. Therefore, this potential selection bias should be considered when interpreting the pathological response and survival outcomes. Second, the relatively small sample size and limited number of outcome events reduced the statistical power for some subgroup analyses, may have limited the identification of additional independent prognostic factors, and increased the risk of model instability despite efforts to minimize overfitting. This concern is particularly relevant to the pathological response analysis, in which only 14 patients achieved a major pathological response. Accordingly, the multivariable logistic regression findings should be interpreted cautiously and require validation in larger cohorts. Third, treatment allocation and administration of postoperative chemotherapy were determined according to routine clinical practice rather than a standardized protocol, introducing the potential for unmeasured confounding. Therefore, any observed association between receipt of adjuvant chemotherapy and survival should not be interpreted as evidence of a causal treatment effect. In addition, detailed data regarding treatment exposure, postoperative complications, and treatment-related toxicity were not consistently available, precluding further analyses of their potential impact on postoperative treatment and long-term outcomes. Finally, although BMI emerged as an independent factor associated with major pathological response, more comprehensive assessments of nutritional status and body composition, such as sarcopenia or muscle mass, as well as formal geriatric assessment tools (e.g., G8 or CARG), comorbidity indices, and the potential influence of molecular characteristics such as HER2 and mismatch repair/MSI status could not be comprehensively evaluated because of the retrospective study design and the limited number of patients with these molecular subtypes.

Nevertheless, our study has several important strengths. To our knowledge, it represents one of the few studies specifically evaluating both major pathological response and long-term survival outcomes in an exclusively older cohort (aged ≥65 years) treated with contemporary perioperative FLOT chemotherapy. Furthermore, the availability of detailed clinicopathological data enabled a comprehensive evaluation of both pretreatment and postoperative prognostic factors, providing clinically relevant information for patient selection and risk stratification in older patients.

In conclusion, our study demonstrates that baseline ECOG PS is independently associated with long-term survival, whereas BMI is independently associated with a major pathological response in older patients with resectable gastric or gastroesophageal junction cancer treated with perioperative FLOT chemotherapy. Furthermore, ypN stage and surgical margin status provided additional prognostic information following surgery. Collectively, these results underscore the importance of comprehensive pretreatment functional and nutritional assessment when considering perioperative FLOT chemotherapy in older patients. Given the retrospective, single-center design and limited sample size, the observed associations should be considered hypothesis-generating and require validation in larger, multicenter prospective cohorts incorporating comprehensive geriatric assessment and objective measures of body composition.

## Figures and Tables

**Table 1:** Baseline clinicopathological characteristics according to major (TRG 0–1) and minor (TRG 2–3) pathological response following neoadjuvant FLOT.

Characteristic	Overall (*n* = 86)	Major Pathological Response (*n* = 14)	Minor Pathological Response (*n* = 72)	*p*
Age group (years), *n* (%)				0.06
65–69	48 (56)	11 (79)	37 (51)	
≥70	38 (44)	3 (21)	35 (49)	
Gender, *n* (%)				**0.04**
Male	62 (72)	7 (50)	55 (76)	
Female	24 (28)	7 (50)	17 (24)	
ECOG PS, *n* (%)				0.17
0	35 (41)	8 (57)	27 (37)	
≥1	51 (59)	6 (43)	45 (63)	
BMI (kg/m^2^), *n* (%)				**0.02**
<25	36 (42)	2 (14)	34 (47)	
≥25	50 (58)	12 (86)	38 (53)	
Location, *n* (%)				**0.04**
Cardia	31 (36)	6 (43)	25 (35)	
Corpus	16 (19)	0 (0)	16 (22)	
Antrum	13 (15)	5 (36)	8 (11)	
Multiple	26 (30)	3 (21)	23 (32)	
Histological type, *n* (%)				0.19
Adenocarcinoma	78 (91)	14 (100)	64 (89)	
Other	8 (9)	0 (0)	8 (11)	
cT stage, *n* (%)				**0.002**
1–2	7 (8)	4 (29)	3 (4)	
3–4	79 (92)	10 (71)	69 (96)	
cN stage, *n* (%)				0.85
N0	11 (13)	2 (14)	9 (12)	
N+	75 (87)	12 (86)	63 (88)	
Clinical stage, *n* (%)				**0.03**
I–II	18 (21)	6 (43)	12 (17)	
III	68 (79)	8 (57)	60 (83)	
Grade, *n* (%)				0.06
1–2	36 (42)	9 (64)	27 (37)	
3	50 (58)	5 (36)	45 (63)	
CEA (ng/mL), *n* (%)				0.42
<5	54 (63)	7 (50)	47 (65)	
≥5	25 (29)	5 (36)	20 (28)	
Missing	7 (8)	2 (14)	5 (7)	
HER2 status, *n* (%)				0.16
Negative	72 (84)	13 (93)	59 (82)	
Positive	9 (10)	0 (0)	9 (12)	
Missing	5 (6)	1 (7)	4 (6)	
Microsatellite status, *n* (%)				0.52
Low	71 (83)	10 (72)	61 (85)	
High	9 (10)	2 (14)	7 (10)	
Missing	6 (7)	2 (14)	4 (5)	

Major pathological response was defined as CAP tumor regression grade (TRG) 0–1 and minor pathological response as CAP TRG 2–3. Bold values indicate statistical significance (*p* < 0.05). *p* values for CEA, HER2, and MSI status were calculated after excluding patients with missing data. Abbreviations: BMI, body mass index; CEA, carcinoembryonic antigen; cN, clinical N stage; cT, clinical T stage; ECOG PS, Eastern Cooperative Oncology Group performance status; HER2, human epidermal growth factor receptor 2; MSI, microsatellite instability.

**Table 2:** Treatment-related and postoperative pathological characteristics according to major (TRG 0–1) and minor (TRG 2–3) pathological response following neoadjuvant FLOT.

Characteristic	Overall (*n* = 86)	Major Pathological Response (*n* = 14)	Minor Pathological Response (*n* = 72)	*p*
ypT stage, *n* (%)				**<0.001**
0–2	30 (35)	12 (86)	18 (25)	
3–4	56 (65)	2 (14)	54 (75)	
ypN stage, *n* (%)				**0.001**
0–1	53 (62)	14 (100)	39 (54)	
2–3	33 (38)	0 (0)	33 (46)	
yp stage, *n* (%)				**<0.001**
0–I	24 (28)	12 (86)	12 (17)	
II–III	62 (72)	2 (14)	60 (83)	
LVI, *n* (%)				**<0.001**
Yes	52 (61)	0 (0)	52 (72)	
No	34 (39)	14 (100)	20 (28)	
PNI, *n* (%)				**<0.001**
Yes	39 (45)	0 (0)	39 (54)	
No	47 (55)	14 (100)	33 (46)	
Surgical margin status, *n* (%)				**<0.001**
R0	70 (81)	14 (100)	56 (78)	
R1–R2	16 (19)	0 (0)	16 (22)	
Adjuvant chemotherapy, *n* (%)				0.19
Yes	69 (80)	13 (93)	56 (78)	
No	17 (20)	1 (7)	16 (22)	

Major pathological response was defined as CAP tumor regression grade (TRG) 0–1, and minor pathological response as CAP TRG 2–3. Bold values indicate statistical significance (*p* < 0.05). Abbreviations: CAP, College of American Pathologists; LVI, lymphovascular invasion; PNI, perineural invasion; TRG, tumor regression grade; ypN, post-neoadjuvant pathological N stage; ypT, post-neoadjuvant pathological T stage.

**Table 3:** Univariable and multivariable logistic regression analyses of factors associated with major pathological response (TRG 0–1).

Characteristic	Univariable Analysis		Multivariable Analysis	
	OR (95% CI)	*p*	OR (95% CI)	*p*
Age group (65–69 vs. ≥70)	3.47 (0.89–13.48)	0.07		
Gender (female vs. male)	3.23 (0.99–10.53)	0.05		
ECOG PS (0 vs. ≥1)	2.22 (0.70–7.10)	0.18		
BMI (≥25 vs. <25)	5.37 (1.12–25.72)	**0.04**	5.13 (1.05–25.19)	**0.04**
Localization (proximal vs. non-proximal/distal)	1.13 (0.35–3.58)	0.84		
Signet-cell histology (no vs. yes)	4.33 (0.53–35.48)	0.17		
Clinical stage (I–II vs. III)	3.75 (1.10–12.79)	**0.03**	3.54 (0.99–12.69)	0.05
Grade (1–2 vs. 3)	3.00 (0.91–9.89)	0.07		

Major pathological response was defined as CAP tumor regression grade (TRG) 0–1. Variables with *p* < 0.05 in the univariable logistic regression analysis were included in the multivariable logistic regression model. Bold values indicate statistical significance (*p* < 0.05). Abbreviations: BMI, body mass index; CAP, College of American Pathologists; CI, confidence interval; ECOG PS, Eastern Cooperative Oncology Group performance status; OR, odds ratio; TRG, tumor regression grade.

**Table 4:** Univariable and multivariable Cox proportional hazards regression analyses for disease-free survival.

Characteristic	Univariable Analysis		Multivariable Analysis	
	HR (95% CI)	*p*	HR (95% CI)	*p*
Age group (≥70 vs. 65–69)	1.95 (1.03–3.69)	**0.04**	1.39 (0.73–2.65)	0.31
Gender (female vs. male)	1.33 (0.68–2.60)	0.40		
ECOG PS (≥1 vs. 0)	3.84 (1.69–8.72)	**0.001**	2.76 (1.19–6.40)	**0.02**
BMI (≥25 vs. <25)	1.85 (0.99–3.48)	0.05		
Localization (proximal vs. non-proximal/distal)	1.70 (0.88–3.29)	0.11		
Signet-cell histology (yes vs. no)	1.33 (0.65–2.74)	0.43		
Clinical stage (III vs. I–II)	1.68 (0.70–4.03)	0.24		
Grade (3 vs. 1–2)	1.84 (0.95–3.54)	0.07		
ypT stage (3–4 vs. 0–2)	2.35 (1.08–5.11)	**0.03**		
ypN stage (2–3 vs. 0–1)	3.82 (1.99–7.31)	**<0.001**	2.58 (1.29–5.17)	**0.008**
Pathological response (minor vs. major)	2.02 (0.72–5.70)	0.18		
LVI (yes vs. no)	2.90 (1.38–6.12)	**0.005**		
PNI (yes vs. no)	2.39 (1.24–4.60)	**0.009**		
Surgical margin status (R1–R2 vs. R0)	3.79 (1.86–7.69)	**<0.001**	1.98 (0.93–4.24)	0.08

Variables included in the multivariable Cox proportional hazards regression models were selected on the basis of statistical significance, clinical relevance, and the number of outcome events to reduce the risk of overfitting. The proportional hazards assumption was assessed using Schoenfeld residuals, and no significant violation was observed (global *p* = 0.65). Bold values indicate statistical significance (*p* < 0.05). Abbreviations: BMI, body mass index; CI, confidence interval; ECOG PS, Eastern Cooperative Oncology Group performance status; HR, hazard ratio; LVI, lymphovascular invasion; PNI, perineural invasion.

**Table 5:** Univariable and multivariable Cox proportional hazards regression analyses for overall survival.

Characteristic	Univariable Analysis		Multivariable Analysis	
	HR (95% CI)	*p*	HR (95% CI)	*p*
Age group (≥70 vs. 65–69)	2.15 (1.08–4.26)	**0.03**	1.68 (0.85–3.33)	0.14
Gender (female vs. male)	1.09 (0.53–2.24)	0.80		
ECOG PS (≥1 vs. 0)	3.80 (1.57–9.18)	**0.003**	2.52 (1.02–6.23)	**0.045**
BMI (<25 vs. ≥25)	1.74 (0.90–3.40)	0.10		
Localization (proximal vs. non-proximal/distal)	2.16 (1.06–4.43)	**0.03**		
Signet-cell histology (yes vs. no)	1.32 (0.63–2.75)	0.46		
Clinical stage (III vs. I–II)	1.79 (0.69–4.64)	0.23		
Grade (3 vs. 1–2)	2.01 (1.01–4.02)	**0.048**		
ypT stage (3–4 vs. 0–2)	2.17 (0.95–4.98)	0.07		
ypN stage (2–3 vs. 0–1)	3.34 (1.68–6.66)	**<0.001**	2.02 (0.97–4.22)	0.06
Pathological response (minor vs. major)	1.59 (0.56–4.52)	0.38		
LVI (yes vs. no)	2.61 (1.18–5.75)	**0.02**		
PNI (yes vs. no)	2.21 (1.10–4.42)	**0.02**		
Surgical margin status (R1–R2 vs. R0)	4.45 (2.13–9.29)	**<0.001**	2.62 (1.20–5.76)	**0.02**

Variables included in the multivariable Cox proportional hazards regression models were selected on the basis of statistical significance, clinical relevance, and the number of outcome events to reduce the risk of overfitting. The proportional hazards assumption was assessed using Schoenfeld residuals, and no significant violation was observed (global *p* = 0.335). Bold values indicate statistical significance (*p* < 0.05). Abbreviations: BMI, body mass index; CI, confidence interval; ECOG PS, Eastern Cooperative Oncology Group performance status; HR, hazard ratio; LVI, lymphovascular invasion; PNI, perineural invasion.

## Data Availability

The data presented in this study are available from the corresponding author upon reasonable request. The data are not publicly available because they contain anonymized patient information and are subject to institutional ethical and privacy restrictions.
